# Outbreak Response to Circulating Vaccine-Derived Poliovirus in Three Northern Regions of Ghana, 2019

**DOI:** 10.1155/2024/5515777

**Published:** 2024-10-04

**Authors:** John Kofi Odoom, Emmanuel Kofi Dzotse, Nicholas Israel Nii-Trebi, David Opare, Ernest Akyereko, Keren Attiku, Ewurabena Oduma Duker, Miriam Eshun, Bismarck Banahene Boahene, Emmanuel Gberbi, Ekua Essumanma Houphouet, Stanley Diamenu, Michael Adjabeng, Joseph Asamoah-Frimpong, Donne Ameme, Joseph Kojo Larbi Opare, Evangeline Obodai

**Affiliations:** ^1^Department of Virology, Noguchi Memorial Institute for Medical Research, College of Health Sciences, University of Ghana, Accra, Ghana; ^2^Disease Surveillance Department, Ghana Health Service, Ministry of Health, Accra, Ghana; ^3^Department of Medical Laboratory Sciences, School of Biomedical and Allied Health Sciences, University of Ghana, Accra, Ghana; ^4^National Public Health and Reference Laboratory, Ghana Health Service, Ministry of Health, Korle-Bu, Accra, Ghana; ^5^World Health Organization, Country Office, Accra, Ghana; ^6^African Field Epidemiology Network, Accra, Ghana; ^7^Ghana Field Epidemiology and Laboratory Training Program, Accra, Ghana; ^8^Ghana Health Service, Private Mail Bag, Ministries, Accra, Ghana

**Keywords:** acute flaccid paralysis (AFP), circulating vaccine-derived poliovirus (cVDPV), environmental surveillance, northern Ghana, outbreak investigation

## Abstract

**Background:** Circulating Vaccine-Derived Poliovirus Type 2 (cVDPV2) was isolated in sewage and later in stool samples from children with acute flaccid paralysis (AFP) in northern Ghana.

**Method:** A multidisciplinary and multisectoral team investigated this outbreak and reported on epidemiological and laboratory investigations. Sewage/wastewater samples were collected from the environment, while stool samples were collected from AFP/contact children under 5 years of age. The samples were processed for virus isolation, and positive isolates were sequenced. We also conducted a descriptive investigation involving a review of records, active case search, and Monovalent Oral Polio Vaccine 2 campaigns. Additionally, we interviewed caregivers about the vaccination status of their children, as well as their knowledge on polio prevention. Water quality, sanitation, hygiene practices, and health-seeking behaviours were also assessed.

**Results:** A total of 18 cVDPV2 were confirmed in the three regions of Ghana during the outbreak in 2019–2020. All strains were genetically linked to a Nigerian cVDPV2 strain NIE-KWS-KSB-18-006HC29 that circulated in 2018. Evaluation of the surveillance system shows that officers have good knowledge of AFP and know how to collect samples, package them, and ship them to the laboratory. Few communities had access to potable water. Open defecation was common, and the water supply, sanitation, and hygiene practices of the communities were poor.

**Conclusion:** The cVDPV2 outbreak represents the first time cVDPV2 has circulated in the country since Ghana embarked on the polio eradication program in 1996. However, with quality mOPV2 mop-up campaigns, a nationwide IPV catch-up campaign coupled with enhanced surveillance measures, transmission was interrupted.

**Summary**

What is already known on this topic?

• cVDPV2 outbreak is already known.
• cVDPV2 has occurred in many countries in Africa.

What this study adds?

• This document reports the first outbreak of cVDPV2 in Ghana since the introduction of AFP surveillance in Ghana in 1996.
• The rapid response of the outbreak to interrupt transmission.

## 1. Introduction

The Global Polio Eradication Initiative (GPEI) strategies, based on the extensive use of the live attenuated oral polio vaccine (OPV), active surveillance, and mop-up, have reduced cases of paralytic poliomyelitis by over 99% [1]. It should be noted that successful eradication of wild poliovirus (WPV) does not necessarily eliminate the risk of paralytic poliomyelitis completely as vaccine-derived poliovirus (VDPV) and vaccine-associated paralytic polio (VAPP) resulting from OPV could cause similar conditions. Furthermore, the main tool for polio eradication, OPV, has been found to be genetically unstable and can revert to neurovirulence during virus replication in the human gut [2–4]. This has led to the genetic drift of OPV to VAPP among recipients of OPV and their susceptible close contacts [2]. Longer replication of the OPV strain has also brought an added risk of emergence and spread of circulating vaccine-derived polioviruses (cVDPVs) in areas where the rate of OPV coverage is low [3]. In countries such as Egypt [5], Hispaniola (Haiti and the Dominican Republic) [6], the Philippines [7], Myanmar and Madagascar [8], Indonesia [9], and China [10], outbreaks of cVDPVs have occurred and been successfully controlled. From 2005 to 2010, Type 2 cVDPV outbreaks were recorded in Nigeria and the Democratic Republic of Congo, Ethiopia, Somalia, and India, while a Type 3 outbreak occurred in Ethiopia from 2009 to 2020. In 2019, the cVDPV outbreak in Nigeria spread to 12 countries in the AFRO region, including Ghana. The ability of the vaccine virus to thrive for several months in sewage treatment plants and in the environment underscores the need to investigate clinical and environmental samples for both WPV and VDPV to help assess the effectiveness of polio eradication strategies [11].

It is noteworthy that environmental surveillance (ES) for polioviruses is now known to be an effective tool for monitoring the circulation of polioviruses in large communities and has been used to detect a number of wild-type and VDPV circulating in areas where AFP surveillance had been on the quiet [12, 13]. The role played by ES continues to be very important, and its sensitivity has been found to be able to detect a strain of poliovirus when one in 10,000 people is excreting the virus [14].

Following the global oral polio vaccination switch, Ghana switched from the use of trivalent oral polio vaccine (tOPV) (consists of vaccine virus Types 1, 2, and 3) to bivalent oral polio vaccine (bOPV) (consists of vaccine virus Types 1 and 3) in April 2016. However, the inactivated polio vaccine (IPV) was not introduced into routine immunization until June 2018 due to global shortages. In the year 2018, Ghana established 10 environmental sites for poliovirus surveillance to complement AFP surveillance after a successful pilot in 2016 [15]. Sewage or wastewater samples are collected from these sites once every 4 weeks and transported through a courier service to the polio laboratory at the Noguchi Memorial Institute for Medical Research (NMIMR), a World Health Organization (WHO)–accredited laboratory for testing. In one such exercise, VDPV2 was isolated from an environmental sample obtained on June 11, 2019, from the drain at the Koblimahgu ES site in the Tamale Metro of the Northern Region. One month later, the first human case of cVDPV2 was confirmed in the Chereponi District in the North East Region. Subsequently, seven more cases were confirmed, four in the Northern and three in the Savannah regions. All strains were genetically linked to a cVDPV2 isolated from an AFP case in Nigeria in 2018 (NIE-KWS-KSB-18-006 HC29). A multidisciplinary and multisectoral team was assembled to investigate this outbreak and to report on epidemiological and laboratory findings and control efforts. Here, we describe the source and magnitude of the outbreak, as well as the control and preventive efforts in response to the cVDPV2 outbreak in the Northern and Savannah regions of Ghana.

## 2. Methods

### 2.1. Outbreak Settings

**Figure 1:**
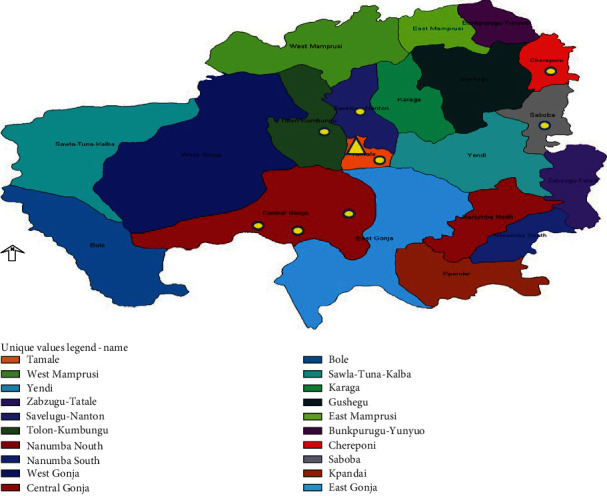
Map of the Northern, Savannah, and North East regions (formerly Northern Region) of Ghana with district locations where cVDPV2 were isolated. The round spot indicates a single cVDPV2 from AFP, while the triangle represents the total cVDPV2 from ES.

The study described here was conducted in the former Northern Region of Ghana, which was split in late 2018 into three regions: the Northern, North East, and Savannah regions (Figure 1). The three regions have an estimated population of 2,479,461 which represents 10.1% of the entire population of Ghana with a land area of 70,384 km^2^. Approximately 45% of the population are children under 15 years old [16]. Due to their proximity to the Sahel and Sahara, the northern regions experience much drier weather conditions than the southern regions of Ghana. Night and day temperatures vary between 14°C (59°F) and 40°C (104°F), respectively. There are more than 500 health facilities, including a teaching hospital, and more than 5000 government health professionals [17]. There are huge markets in these regions where people from all over Ghana and beyond (Burkina, Mali, Niger, Nigeria, Togo, etc.) come to do business. There is also a high movement of the nomadic population from community to community and district to district with their cattle.

**Figure 2:**
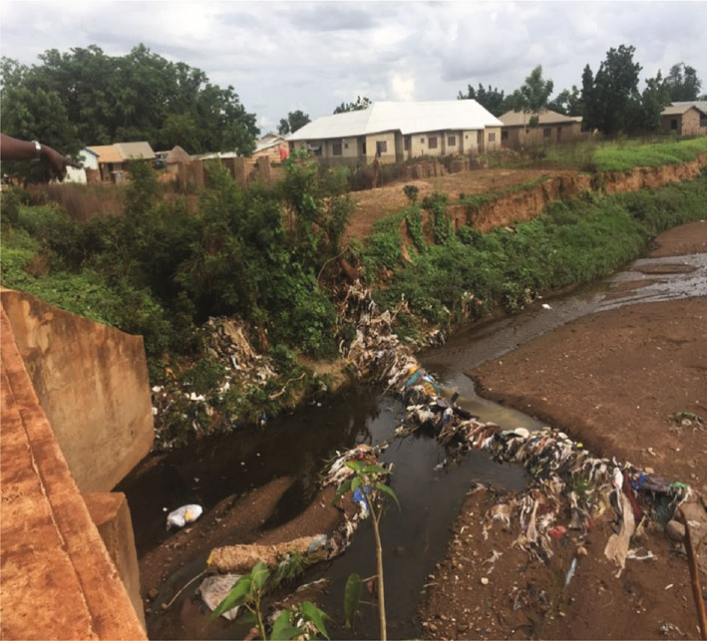
Environmental surveillance site at Koblimahgu in the Tamale Metro District.

The Tamale Metro is one of the most populated places in the Northern Region (Figure 1). There is an ES site present where the recent incident of cVDPV2 was isolated. The site has a large drainage stretching for about 5 km (Figure 2). Sanitation here is substandard, and due to the lack of toilet facilities in some homes, open defecation is common, and residents also discard faecal matter into the available drains. Children play or loiter in the drain, and this increases the likelihood of them coming into contact with faecal matter.

#### 2.1.1. Detection of First cVDPV2 From Sewage

Sewage or wastewater samples are collected from the Northern Region ES site once every 4 weeks and transported through a courier service to the polio laboratory at the NMIMR for testing. On July 8, 2019, a cVDPV2 was confirmed from an environmental sample obtained on June 11, 2019, from the drain in Koblimahgu environmental site. A multisectoral team from the Ghana Health Service (GHS), the WHO, the United States Centers for Disease Control and Prevention (CDC), and the Ghana Field Epidemiology and Laboratory Training Programme (GFELTP) was subsequently deployed on July 10, 2019, to assist the Northern Regional and Tamale Metropolitan Health Directorate to investigate the outbreak.

#### 2.1.2. Detection of the First Human Case of cVDPV2

The Ando-Nyamanu community is in the Chereponi District of the North East Region, where the first human case was diagnosed. It is located in the northeast corridor of the northern part of Ghana. Chereponi is the capital of the district (Figure 1). The population under 5 years is approximately 13,469. The inhabitants are mainly of Anufor (Chokosi) ethnic extraction who have family members throughout northern Togo. There are a few settler farmers, including Ewes, Battors, Moshis, and nomadic Fulanis.

The index case was a female of 31 months. She had an acute onset of fever, cough, and running nose on July 19, 2019. Two days later, she developed weakness in both lower limbs and was taken to the hospital because she was unable to walk the following morning. About 2 weeks before the onset of recent illness, the patient had been treated at an unregistered private facility for unnamed fever-rash disease associated with fluid-filled skin lesions for which she was given an injection of unspecified medication in each buttock. Laboratory results of the patient confirmed malaria and anaemia (HB 8.7 m/dL) on July 25, 2019, and hospitalized at the Chereponi Government Hospital. While on admission, the clinician suspected AFP, and two stool samples were taken 24 h apart and sent to the polio laboratory in NMIMR for testing. The patient was discharged home on Day 4 when the physician assistant attending to her thought she was well enough to go home even though the child could not still stand up and walk. A review was scheduled in 2 weeks by which results of stool specimens might have been received. When the polio laboratory reported a cVDPV, an outbreak investigation team was constituted to visit the child and the community to conduct an investigation.

### 2.2. Study Design

In August 2019, a team of GHS staff and health partners visited the Regional Health Directorate of the Northern, North East, and Savannah regions and their respective regional, district, and other health facilities where cases had been detected. The visiting and local health teams conducted desk reviews of relevant available health reports, including AFP surveillance operations and immunization evaluation, interviewed relevant stakeholders, reviewed patients (clients) outpatient department (OPD) and admission records, and performed an active case search for AFP cases in the regions. Case patients were clinically examined. The community water, sanitation, and hygiene (WASH) assessment was carried out, and the social mobilization and risk communication evaluation was also carried out. Furthermore, the team assessed vaccine cold chain and logistic management, including temperature monitoring, as well as immunisation data management and coverage.

### 2.3. Virus Isolation and Intratypic Differentiation

A liter of sewage was received in the polio laboratory on June 12, 2019, 1 day after collection at the Komblimahgu ES site. Five hundred milliliters of sewage was concentrated by the two-phase separation method. Briefly, 500 mL of sewage was transferred into a centrifuge tube and centrifuged at 1000 × g for 20 min at 4°C. The supernatant was carefully poured into a 1 L beaker, and the pH of the sample was adjusted to 7–7.4 using a pH strip or 1 N NaOH. Two hundred eighty-seven milliliters of 29% PEG, 39.5 mL of 22% dextran, and 35 mL of 5 N NaCl were added to the samples and mixed thoroughly by vigorous stirring for 1 h on a magnetic stirrer. During stirring, the burette stand was prepared and used to hang the separation funnel in a vertical upright position. The upper and lower stoppers of the funnel were greased. The funnel valve was checked for leakage by pouring a few milliliters of sterile distilled water. The sample was poured into the separation funnel carefully, and the funnel was left to stand overnight at 4°C. The phase separation was checked, and the entire lower phase and the interphase were harvested in a dropwise pattern into a sterile 50-mL centrifuge tube. Sewage concentrates and AFP samples were treated with chloroform and inoculated on rhabdomyosarcoma (RD) and mouse L cells (L20B) following standard protocols [18]. Cultured polioviruses were further characterized by reverse transcriptase polymerase chain reaction (RT-PCR) assays using primer sets specifically designed to detect enterovirus and poliovirus group, serotype, and Sabin strains [19].

### 2.4. Sequencing of the First ES and AFP Samples and Data Analysis

VDPVs were identified by VP1 sequencing [20]. Sequence files including raw data, consensus, and contig sequences were sent to the Center for Disease Control and Prevention, Atlanta, United States, to determine the closest match and final classification of the VDPV2. Phylogenetic relationships between strains were established by comparing sequences obtained with reference sequences through alignment with the CLUSTAL X alignment program implemented in the MEGA 4.0 software package [21, 22]. Phylogenetic relationships between sequences were inferred by the maximum likelihood method with DNADIST/NEIGHBOR of PHILIP (phylogenetic inference package) Version 3.6 software [23]. The robustness of phylogenies was estimated by bootstrap analysis with a 1000 pseudoreplicate data set generated with the SEQBOOT program of PHILIP. A phylogenetic tree was constructed using the neighbor-joining feature of PHILIP and drawn using TREEVIEW software [24].

## 3. Results

### 3.1. Environmental Assessment

The sampling site is a large open drain that passes through more than 10 communities in the Tamale Metropolitan Area and beyond. It has water flowing through some parts and parts filled with litter. There are settlements at the edges of the drain without waste bins. Pouring of liquid, solid, and most importantly, human waste into the main drainage was common. The environment along the drainage was bushy, creating an enabling environment for open defecation, especially by children. Children played or loitered in the drain, and this increases the likelihood of them coming into contact with faecal matter. Faecal matter could be seen along the walk paths around the drain. The environmental health assessment of Chereponi indicated that the community has access to potable water in the form of mechanised borehole. In general, WASH practices by the community were fairly good.

### 3.2. Assessment of the AFP Surveillance System

AFP surveillance, though not optimal, was ongoing in the Northern, North East, and Savannah regions and ES in the Tamale Metropolitan Area. All health facilities visited had focal persons with assistants, who had served for more than a year, responsible for public health surveillance. At least one focal person had been formally trained in public health surveillance that addressed AFP. Some focal persons had a supervisory visit from the Metropolitan Health Directorate in the last 6 months. All but one of the officers at Tamale Central Hospital had good knowledge of the definition of AFP cases, two of whom had an appreciable understanding of proper sample collection, packaging and transportation, and stool adequacy. Only one mentioned the 60-day follow-up as part of the AFP surveillance and case investigation. The nurses in charge of the various units, namely, the pediatric ward, OPD, emergency, and adult male and female wards, did not know about AFP surveillance. The reviewed records also showed that Tamale West Hospital had identified and reported two AFP cases in 2019 and two in 2018. In all facilities visited, the review of the records did not identify any case of missed AFP. AFP posters were found only on the walls of Tamale West Hospital with the appropriate contact number for reporting. The SDA hospital had a poster of the reportable priority diseases posted in the disease control department.

Hospital surveillance record review was irregular within the Chereponi Health Directorate. Although there was some evidence of records review, further probing revealed it was done retrospectively. Of the 6350 case records we reviewed, including 1053 records from the A&D register, 31 inpatient records (A&D register) had no record of the diagnosis. We did not identify any missed AFP within the period under review. Although the case of AFP was recorded in the OPD register, there was no corresponding record in the A&D register in spite of the patient being admitted.

### 3.3. Immunization Performance

OPV immunization coverage (administrative coverage) for the region and Tamale Metro is above 100%, as shown in Table 1; however, the 2017 EPI coverage survey shows lower coverage, less than 80%. There is a challenge in the quality of the immunization data with respect to target populations. There have not been any unimmunised (missed) children over the years since the administrative coverage indicates more children have been immunized than targeted (Table 1).

Whilst the OPV1/3 dropout rate for the region shows a decline in trend and that of the Tamale Metro shows an increasing trend, the 2018 dropout rate for the metro showed an increase over that of 2017. The dropout rate for the Vittin submetro, where the ES site is located, so far for 2019 was 8%, an increase over the 2018 figure of 6.5%.

There have been high coverages (over 100%) for all NIDs from 2012 to 2015 for the region and the Tamale Metro. As shown in Table 1, the Tamale Metro was not part of the selected districts for the NID in 2015.

The cold chain was well maintained at the district vaccine store and the Chereponi Health Centre, with temperatures within the recommended range of +2°C–+8°C. Vaccination monitoring was good with appropriate temperature charting and up-to-date coverage monitoring. Of 34 children surveyed in the community, 33 had vaccination cards, giving a card retention rate of 97%. The only child who did not possess a vaccination record book or card had had the card burned during the recent ethnic conflict. As such, the record was excluded from the final assessment. OPV3 coverage was 94% (31/33), while of the 12 who were eligible for IPV, 6 (50%) were vaccinated. There was a correspondingly high coverage of OPV3 and relatively low coverage of IPV from the administrative data. No additional AFP cases were detected in the community. Caregivers indicated that immunization was lifesaving, but just about half of them knew the cause, transmission, and prevention of polio, and only a handful knew more than one disease preventable by immunization. The main sources of information on vaccination are health workers and community leaders/volunteers.

### 3.4. Risk Communication Rapid Assessment

Caregivers report they were not informed about the vaccination their children were given at the child welfare clinics. There were poor waste disposal habits, which resulted in the poor sanitation of the communities. Most of the houses had no waste bins, even at the final waste disposal site. Pouring of liquid, solid, and most importantly, human waste into the main drainage was common. The environment along the drainage was bushy, creating an enabling environment for open defecation.

Of the 16 caregivers interviewed, only four responded they rely on pipe-borne water and two rely on wells and other sources. Two of the caregivers had their own toilet, while the remaining 14 responded they use the public KVIP and open defecation at night. Children, on the other hand, do not use the KVIP but defecate close to the drain. The majority (15/16) responded they wash their hands with soap and water after using the toilet.

### 3.5. Assessment of the Local Capacity to Respond to the Outbreak

The regions have a Public Health Emergency Management Committee (PHEMC) in place to allow them to respond to outbreaks, but the committee was inactive. A database of trained district public health emergency rapid response teams (PHERRTs) was available; however, no database was available for the regional PHERRT.

Tools for emergency preparedness and response activities, including integrated disease surveillance and response (IDSR) technical guidelines and standard operating procedures (SOPs) for meningitis and cholera, were available. There were no tools specific to the response to the polio outbreak.

### 3.6. Laboratory Analysis

**Figure 3:**
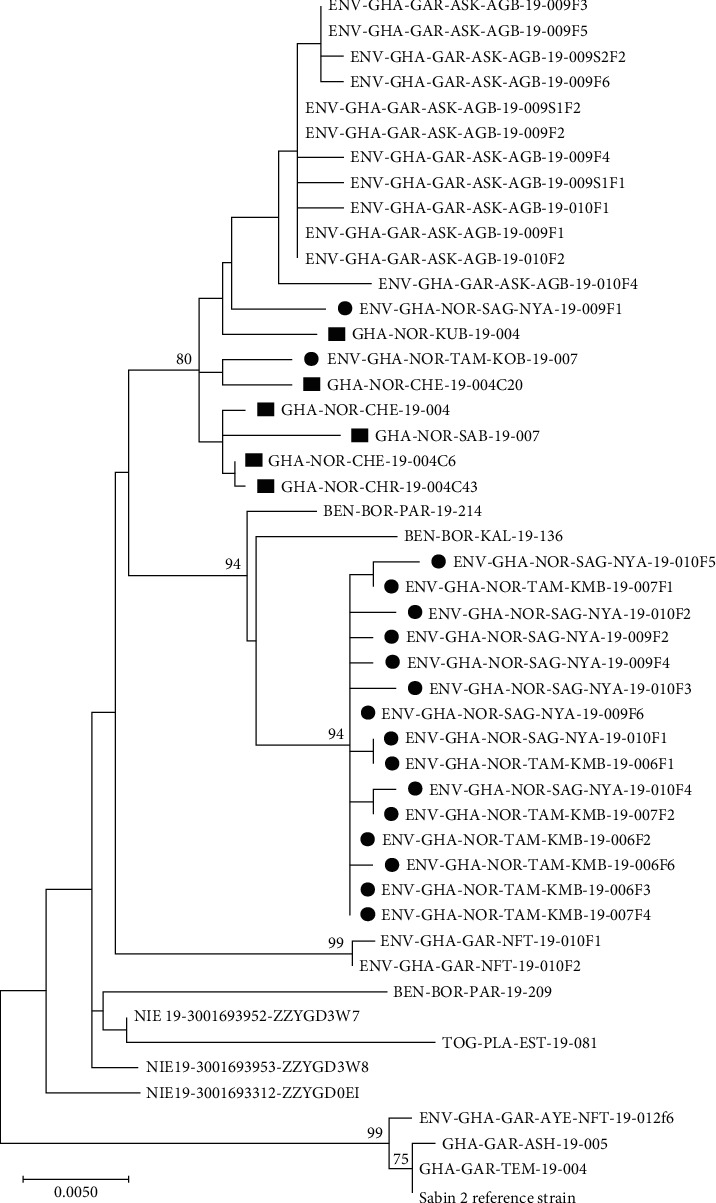
Phylogenetic analysis of cVDPV2 that circulated in the Northern Region in the 2019 Ghana outbreak. Black squares indicate cVDPV2 from AFP and contacts. Black circles represent cVDPV2 from environmental samples. “F” at the end of the environmental isolate Epid number represents the flask number that was positive.

A total of 18 cVDPV2 were confirmed during the outbreak. Five cVDPV2 samples from sewage and four from AFP were identified and confirmed in the Northern Region. Within the North East Region, an AFP case with three contacts was confirmed, and in the Savannah Region, two cases and three contacts were confirmed. All strains were genetically linked to a Nigerian cVDPV2 strain NIE-KWS-KSB-18-006HC29 that circulated in 2018. All affected children were born after the switch from tOPV to bOPV polio vaccination and had received some doses of bOPV but had no history of OPV containing Type 2 or IPV (Table 2). The closest match to the isolate was determined by constructing a phylogenetic analysis with more recent cVDPVs. As shown in Figure 3, all 18 cVDPV2 were grouped into two groups. The first cluster comprises AFP and contacts isolates from Chereponi, Saboba, and Kumbugu that are related to each other and linked to two environmental isolates from Koblimahgu and Nyanshegu. The second cluster is made up of the remaining environmental isolates from Koblimahgu and Nyanshegu that are phylogenetically close to each other and distantly linked to a Benin AFP isolate that also circulated in 2019.

### 3.7. cVDPV2 Outbreak in the North East and Northern Regions

Sequence analysis of isolates of the AFP index case from Chereponi identified a 26-nucleotide difference from Sabin 2, while three isolates from its contacts showed a 25-, 27-, and 32-nucleotide difference from Sabin 2. These findings indicate undetected virus circulation for at least 2 years (Table 2). The cVDPV2 cases from Saboba and Kumbugu in the Northern Region with the dates of onset of paralysis on August 30, 2019, and September 24, 2019, had a 27 and 25 nucleotide difference with 97% and 97.2% homology to Sabin 2 (Table 2). The cVDPV2 strains from the Tamale and Savelugu districts also in the Northern Region had a 28-nucleotide difference from Sabin 2. All cases and contacts were clustered with the NIE-JS-1 emergence group.

### 3.8. cVDPV2 Outbreak in the Savannah Region

Three cases of cVDPV2 were reported from the central Gonja district of the Savannah Region from October 7 to November 11, 2019. The first case of paralysis associated with this outbreak occurred on October 7, 2019. The VDPV2 isolate of that case had a 30-nucleotide difference from Sabin 2, which might suggest nearly 2 years of undetected circulation. Subsequently, two additional cases were reported that involved genetically linked viruses with the onset of paralysis on October 28, 2019, and November 18, 2019.

## 4. Discussion

AFP represents the gold standard and therefore a key factor in efforts to achieve a successful eradication of poliomyelitis. Between July and September 2019, eight cVDPV2 outbreaks were reported in six districts in the Northern, North East, and Savannah regions of Ghana. Four of the cases occurred in the Northern Region, one occurred in the North East Region, and three in the Savannah Region.

The single emergence of cVDPV was first detected by ES in Koblimahgu in the Tamale Metro in July 2019 and was later detected in AFP cases in other districts. The Tamale Metro outbreak was the first cVDPV outbreak since the country joined the polio eradication initiative in 1996. This finding might suggest that low levels of vaccination coverage, as well as gaps in AFP surveillance, could have resulted in prolonged, undetected circulation of cVDPV2 in the community for up to 3 years before the virus was detected in the drain [6]. The introduction of ES in Ghana with the sampling of wastewater or wastewater in some selected regions where clinical cases had been reported underscored the supplementary value of ES as an effective approach for the surveillance of AFP, especially when monitoring the silent circulation of poliovirus within a population.

Ghana was declared a polio-free country by the Regional Polio Certification Committee in 2015 [25]. Since then, Ghana has continuously achieved above 95% coverage of OPV3 through routine immunisation and ensured some quality in AFP surveillance. Despite the strides made towards global eradication, inherent transmission persists in some areas in Africa and Asia. Therefore, the possibility of importing WPV into polio-free countries can never be underestimated [26].

OPV vaccination has played an important role in the elimination of WPV. OPV contains attenuated (weakened) but transmissible viruses that can spread from one person to another. In its attenuated form, interindividual spread is beneficial as it contributes to population immunity. However, the attenuation of OPV is unstable and, in rare instances, can revert to virulent form, which causes vaccine-derived paralytic poliomyelitis outbreaks. As such, OPV serves not only as a vaccine but also as a source of poliovirus. Therefore, to complete the eradication of poliomyelitis, its use in vaccination must be discontinued.

In September 2015, the Global Commission for the Certification of Poliomyelitis Eradication declared the eradication of Wild Type 2 Poliovirus (WPV2) worldwide. The commission further recommended that the global synchronized “switch” from the use of tOPV to bOPV should take place in April 2016 [27]. The Type 2 component of tOPV was responsible for > 90% of cVDPV cases recorded during the years 2006–2015. The last case of Natural-Occurring WPV2 observed was in India in 1999 [28]. In addition to OPV, Sabin strain viruses are also capable of transmission, which can circulate in low-immunity settings, constituting cVDPV lineages [29]. It should be noted that among the three OPV serotypes, OPV2 is estimated to cause 40% of all cases associated with VAPP and 90% of all cVDPV cases before 2016 [30]. Therefore, the successful removal of OPV2 from preferential use confers some public health benefits. However, stopping OPV2 immunization may constitute a risk that Sabin strain lineages survive to become cVDPV2 in the future, which will require a response to the outbreak with OPV2 that could potentially generate new lineages [29].

A single dose of IPV comprising the three poliovirus serotypes was introduced into routine immunization schedules in countries that use OPV to reduce the risk of a gap in immunity to Poliovirus Type 2 (PV2) [26, 31]. Ghana switched from using tOPV to bOPV on April 14, 2016, but due to a global IPV shortage, IPV was not introduced until June 2018 [32]. Therefore, there is a high possibility of transmission of polio in any part of Ghana for the following reasons, which may account for the outbreak of polio in Chereponi and other places in Ghana. First, there are cohorts of children in the country who are naïve to the Sabin 2 virus. These were born after the switch from tOPV to bOPV in April 2016, and, as such, they are at a very high risk of contracting poliomyelitis due to PV2. Second, inadequate review of surveillance documentation and records coupled with inadequate community knowledge on causes, transmission, and prevention of polio, as well as free movement across the border with neighbouring Togo and other countries, make the district a high risk of transmission. At the time of the investigation, there was an ethnic conflict, and a curfew was imposed between 6 pm and 6 am with police and military presence to ensure the maintenance of law and order.

Prior to the present finding, cVDPV had previously been isolated in the last 30 days at an ES site in Tamale. The determination of the closest match was related to the 2018 Nigerian cVDPV strain NIE-KWS-KSB-18-006HC29 isolated from an AFP case with 6 nucleotide differences (99.34% similarity) [33]. In the outbreak investigations, it was realized that the Ando-Nyamanu community has access to potable water in the form of a mechanized well. In general, the WASH practices of the community were fairly good. The introduction of IPV in routine immunization worldwide between 2014 and 2016 was intended to protect against poliomyelitis among children born after OPV cessation [34]. However, without appreciable improvements in sanitation, sustained IPV supply [35], and routine immunization coverage, IPV alone may not be sufficient to protect against poliovirus circulation in all settings.

Our findings indicated that the analysis of the sequences derived from the VP1 region of the genome of all cVDPV2 from both sewage and humans (patients and contacts) indicated a genetic link to VDPV2 that circulated in Nigeria in 2018 (NIE-KWS-KBS-18-006HC29), which belongs to the NIE-JIS-1 emergence group [33]. This underscores the need for routine ES to detect circulation strains in real time to avoid outbreaks. Situations that fuel the spread of the virus, such as open defecation and lack of pipe-borne water in most communities, also need to be addressed. Surveillance gaps including difficulty in reaching and monitoring remote or hard-to-reach areas could be overcome by the use of innovative approaches like mobile health teams or satellite surveillance to increase access to remote areas. Similarly, incomplete data, making it difficult to track progress, can also be minimized by implementing data quality checks and provision of training to improve data quality. Importantly, the coverage of the polio vaccine in these regions appears to be high over the past 5 years, as per the record review. However, our findings of cVDPV2 that have been in circulation for more than 2 years without detection suggest that there were immunization gaps in some districts in the Northern, North East, and Savannah regions. Other gaps identified were low vaccination rates in certain areas or populations and uneven distribution of vaccines, leaving some areas or groups underserved. Targeted interventions like focusing on underserved areas and populations and strengthening cold chain management through investing in proper storage and handling infrastructure can help minimise future outbreaks.

## 5. Conclusion

The detection of VDPV2 in both sewage and AFP was the first documentation of the circulation of VDPV2 in the country. Effective mOPV2 mop-up campaigns, a nationwide IPV catch-up campaign coupled with enhanced surveillance activities, were implemented to interrupt transmission. Measures to strengthen surveillance with high routine and additional immunization activities have since been implemented to prevent the future emergence and spread of VDPV in the country.

## Figures and Tables

**Table 1:** Tamale Metro immunization coverage (%) 2013–2019.

**Antigen**	**Year**				
	**2015**	**2016**	**2017**	**2018**	**2019**
Target	10,302	10,601	10,909	10,909	11,022
OPV1	18,074	17,985	17,698	22,527	9523
OPV3	16,710	15,866	16,369	20,255	9029
IPV				8098	8844
OPV 1 (%)	175	170	162	207	86
OPV 3 (%)	162	150	150	186	82
OPV1/3(DOR [%])	8	12	8	10	5
IPV (%)				74.2	80.2
Unimmunized	−6408	−5265	−5460	−9346	1993

**Table 2:** Description of AFP cases and contacts associated with Vaccine-Derived Poliovirus Type 2.

**Epid no.**	**Age in months**	**Sex**	**Date of last OPV**	**Onset of paralysis**	**Collection date**	**Percent match Sabin 2**	**Nucleotide diff Sabin 2**
GHA-NOR-CHE-19-004	32	F	14/02/17	23/07/2019	27/07/19	97.24	25
GHA-NOR-SAB-19-007	35	M	4 doses	30/08/2019	08/08/19	97	27
GHA-NOR-KUM-19-004	26	M	26/09/19	24/09/2019	28/09/19	97.2	25
GHA-NOR-TAM-19-00	36	F	Unknown	24/09/2019	10/10/19	96.8	29
GHA-SVN-CGD-19-003	27	M	Unknown	04/10/2019	17/10/19	96.7	30
GHA-NOR-SAV-19-002	76	F	29/10/13	08/10/2019	15/10/19	96.9	28
GHA-SVN-CGD-19-004	24	M	Unknown	23/10/2019	28/10/19	96.8	29
GHA-NOR-CHE-19-004C20^a^	108	M	3 doses	N/A	21/08/19	96.9	28
GHA-NOR-CHE-19-004C6^a^	24	F	2 doses	N/A	21/08/19	97.01	27
GHA-NOR-CHE-19-004C43^a^	45	M	3 doses	N/A	21/08/19	97.23	25
GHA-SVN-CGD-19-003C1^a^	27	M	Unknown	N/A	17/11/19	96.5	32
GHA-SVN-CGD-19-004C2^a^	24	M	Unknown	N/A	14/11/19	96.8	29
GHA-SVN-CGD-19-005C3^a^	18	M	Unknown	N/A	14/11/19	96.9	28

^a^Contacts of cases.

## Data Availability

All the data supporting this study are included in the article.
